# Affinity fine-tuning anti-CAIX CAR-T cells mitigate on-target off-tumor side effects

**DOI:** 10.1186/s12943-024-01952-w

**Published:** 2024-03-16

**Authors:** Yufei Wang, Alicia Buck, Brandon Piel, Luann Zerefa, Nithyassree Murugan, Christian D. Coherd, Andras G. Miklosi, Haraman Johal, Ricardo Nunes Bastos, Kun Huang, Miriam Ficial, Yasmin Nabil Laimon, Sabina Signoretti, Zhou Zhong, Song-My Hoang, Gabriella M. Kastrunes, Marion Grimaud, Atef Fayed, Hsien-Chi Yuan, Quang-De Nguyen, Tran Thai, Elena V. Ivanova, Cloud P. Paweletz, Ming-Ru Wu, Toni K. Choueiri, Jon O. Wee, Gordon J. Freeman, David A. Barbie, Wayne A. Marasco

**Affiliations:** 1 Department of Cancer Immunology and Virology, Dana-Farber Cancer Institute, Boston, MA 02215 USA; 2 Harvard Medical School, Boston, MA 02115 USA; 3 Department of Medical Oncology, Dana-Farber Cancer Institute, Boston, MA 02215 USA; 4 ONI (Oxford Nanoimaging Ltd.), Oxford, UK; 5 Molecular Imaging Core, Dana-Farber Cancer Institute, Boston, MA 02215 USA; 6 Department of Pathology, Brigham and Women’s Hospital, Boston, MA 02115 USA; 7 Department of Oncologic Pathology, Dana-Farber Cancer Institute, Boston, MA 02215 USA; 8 LUMICKS, Waltham, MA 02453 USA; 9 Lurie Family Imaging Center, Center for Biomedical Imaging in Oncology, Dana-Farber Cancer Institute, Boston, MA 02215 USA; 10 Belfer Center of Applied Cancer Science, Dana-Farber Cancer Institute, Boston, MA 02215 USA; 11 Lowe Center for Thoracic Oncology, Dana-Farber Cancer Institute, Boston, MA 02215 USA

**Keywords:** Chimeric antigen receptor (CAR) T, Affinity/avidity fine-tuned, Clear cell renal cell carcinoma (ccRCC), Carbonic anhydrase IX (CAIX), Direct stochastic optical reconstruction microscopy (dSTORM), On-target off-tumor (OTOT) toxicity

## Abstract

One of the major hurdles that has hindered the success of chimeric antigen receptor (CAR) T cell therapies against solid tumors is on-target off-tumor (OTOT) toxicity due to sharing of the same epitopes on normal tissues. To elevate the safety profile of CAR-T cells, an affinity/avidity fine-tuned CAR was designed enabling CAR-T cell activation only in the presence of a highly expressed tumor associated antigen (TAA) but not when recognizing the same antigen at a physiological level on healthy cells. Using direct stochastic optical reconstruction microscopy (dSTORM) which provides single-molecule resolution, and flow cytometry, we identified high carbonic anhydrase IX (CAIX) density on clear cell renal cell carcinoma (ccRCC) patient samples and low-density expression on healthy bile duct tissues. A Tet-On doxycycline-inducible CAIX expressing cell line was established to mimic various CAIX densities, providing coverage from CAIX-high skrc-59 tumor cells to CAIX-low MMNK-1 cholangiocytes. Assessing the killing of CAR-T cells, we demonstrated that low-affinity/high-avidity fine-tuned G9 CAR-T has a wider therapeutic window compared to high-affinity/high-avidity G250 that was used in the first anti-CAIX CAR-T clinical trial but displayed serious OTOT effects. To assess the therapeutic effect of G9 on patient samples, we generated ccRCC patient derived organotypic tumor spheroid (PDOTS) ex vivo cultures and demonstrated that G9 CAR-T cells exhibited superior efficacy, migration and cytokine release in these miniature tumors. Moreover, in an RCC orthotopic mouse model, G9 CAR-T cells showed enhanced tumor control compared to G250. In summary, G9 has successfully mitigated OTOT side effects and in doing so has made CAIX a druggable immunotherapeutic target.

**Supplementary Information:**

The online version contains supplementary material available at 10.1186/s12943-024-01952-w.

## Introduction

Chimeric antigen receptor (CAR) T cell therapy has achieved significant success in the treatment of hematological malignancies [1], however these results have not yet been translated to solid tumors [2]. The most significant challenge of solid tumor CAR-T cell therapy arises from a lack of tumor-specific antigens (TSAs). Most therapeutic targets are tumor-associated antigens (TAAs), that are expressed at low levels on healthy cells (i.e. epidermal growth factor receptor (EGFR) [3], human epidermal growth factor receptor 2 (HER2) [4], mucin 1 (MUC1) [5] and carcinoembryonic antigen (CEA) [6]) leading to on-target off-tumor (OTOT) toxicity due to CAR-T cell targeting of those low TAA expressing cells.

Clear cell renal cell carcinoma (ccRCC) is a major subtype of renal cell carcinoma (RCC), which is among the 10 most common cancers in both men and women [7–9]. Carbonic anhydrase IX (CAIX), a downstream gene product of hyperactivation of the hypoxia inducible factor (HIF) pathway, represents an important therapeutic target for patients with ccRCC [10, 11]. Meanwhile, CAIX is also found in the epithelium of the bile duct and small intestine as well as in mucous cells of the gastric epithelium [12]. In an early anti-CAIX CAR-T clinical trial, Lamers et al. tested a first-generation G250 CAR-T comprised of a single CD3ζ costimulatory domain in metastatic ccRCC patients [13–15]. However, all patients treated with the G250 CAR-T cells developed grade 2–4 liver enzyme disturbances from the recognition of low CAIX expression on healthy bile duct cells [13–15]. In addition to the reported liver toxicities, the clinical trial observed immunogenicity of the murine single chain variable fragment (scFv) domains resulting in limited persistence of the injected CAR-T cells [16]. The three main limitations of this study are summarized: (i) OTOT toxicity; (ii) immunogenicity of the murine G250 CAIX CAR receptor and (iii) the lack of T-cell persistence and therapeutic efficacy [13].

To optimize anti-CAIX CAR-T cell therapy, we engineered an affinity/avidity fine-tuned CAIX targeted CAR with a low-affinity human scFv G9 followed by the 41BB costimulatory domain which has demonstrated superior efficacy and persistence in vivo [17]. G9 CAR-T cells exhibited high avidity against high density CAIX on ccRCC skrc-59 cells and low avidity against CAIX low MMNK-1 cholangiocytes. This signifies successful mitigation of off-tumor killing on normal tissues with physiological expression levels of the tumor antigen. Furthermore, G9 CAR-T cells exhibited superior tumor killing compared to G250 CAR-T cells in an orthotopic ccRCC mouse model.

## Results

### Skrc-59 CAIX+ recapitulates CAIX expression on ccRCC patient samples

**Fig. 1:**
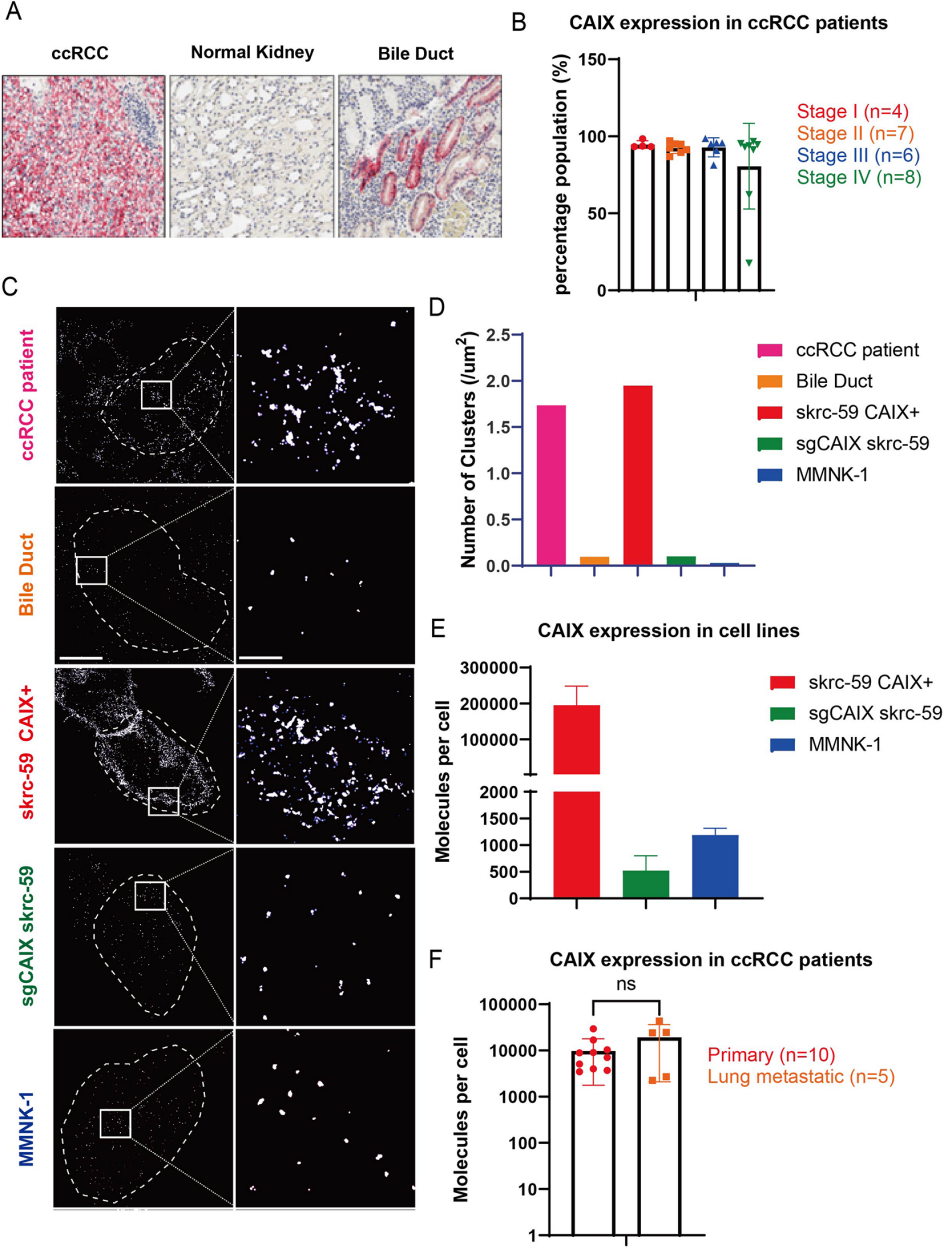
Quantification of CAIX on patient ccRCC samples, healthy bile duct, and cell lines. (**A**) IHC staining of CAIX on ccRCC and bile duct samples. (**B**) CAIX positivity quantified by IHC on ccRCC patient samples from different stages, Stage I (red), Stage II (orange), Stage III (blue) and Stage IV (green). (**C**) High resolution representative images of CAIX on ccRCC (pink), bile duct (orange), skrc-59 CAIX+ cell line (red), sgCAIX skrc-59 cell line (green) and MMNK-1 cell line (blue). (**D**) Bar plot of CAIX expression quantified by dSTORM presented by number of clusters on ccRCC (pink), bile duct (orange), skrc-59 CAIX+ cell line (red), sgCAIX skrc-59 cell line (green) and MMNK-1 cell line (blue). (**E**) Bar plot of CAIX expression quantified by flow cytometry on skrc-59 CAIX+ cell line (red), sgCAIX skrc-59 cell line (green) and MMNK-1 cell line (blue) with mean values of 195,176, 523, 1189 accordingly. (**F**) Bar plot of CAIX expression quantified by flow cytometry on ccRCC patient samples from primary and lung metastatic lesions, primary (red, n = 10), lung metastatic (orange, n = 5), with mean values of 10,396, 19,292 accordingly. All data with error bars are presented as mean ± SD. *P* values are defined by unpaired two-tailed t-tests (*p < 0.05; **p < 0.01; ***p < 0.001; and ****p < 0.0001)

Low expression of CAIX on cholangiocytes [18] (Fig. 1A) leads to OTOT of the previous anti-CAIX CAR-T therapy. However, the high CAIX prevalence in ccRCC, regardless of stage, makes CAIX a promising therapeutic target (Fig. 1B) if OTOT can be mitigated. To better understand CAIX expression on ccRCC tumor and normal bile duct tissue, we collected fresh frozen tissue sections and performed direct stochastic optical reconstruction microscopy (dSTORM) super resolution imaging for CAIX quantification using a fluorophore-conjugated primary anti-CAIX antibody (Fig. 1C). The results showed that the skrc-59 CAIX+ tumor cell line used in our research [17, 19–21] recapitulates CAIX expression levels on ccRCC patient tissue, while both sgCAIX skrc-59 tumor cells, that were engineered to knock out CAIX using CRISPR/Cas9 and single guided RNA (sgRNA) targeting CAIX, and MMNK-1 cholangiocyte [22] cells represent a similar CAIX density as observed in bile duct (Fig. 1D).

Using quantification beads, our analysis showed that MMNK-1 cells have an average of 1,278 CAIX molecules, while ccRCC skrc-59 tumor cells have an average of 207,111 molecules on the cell surface, translating into a circa 200-fold CAIX density difference between tumor and normal tissues (Fig. 1E). Furthermore, quantifying CAIX expression on tumor tissues from primary and lung metastatic lesions of ccRCC demonstrated a similar CAIX expression level (Fig. 1F).

### Tet-On inducible CAIX expressing skrc-59 cell mimics CAIX expression on tumor and healthy cells

**Fig. 2:**
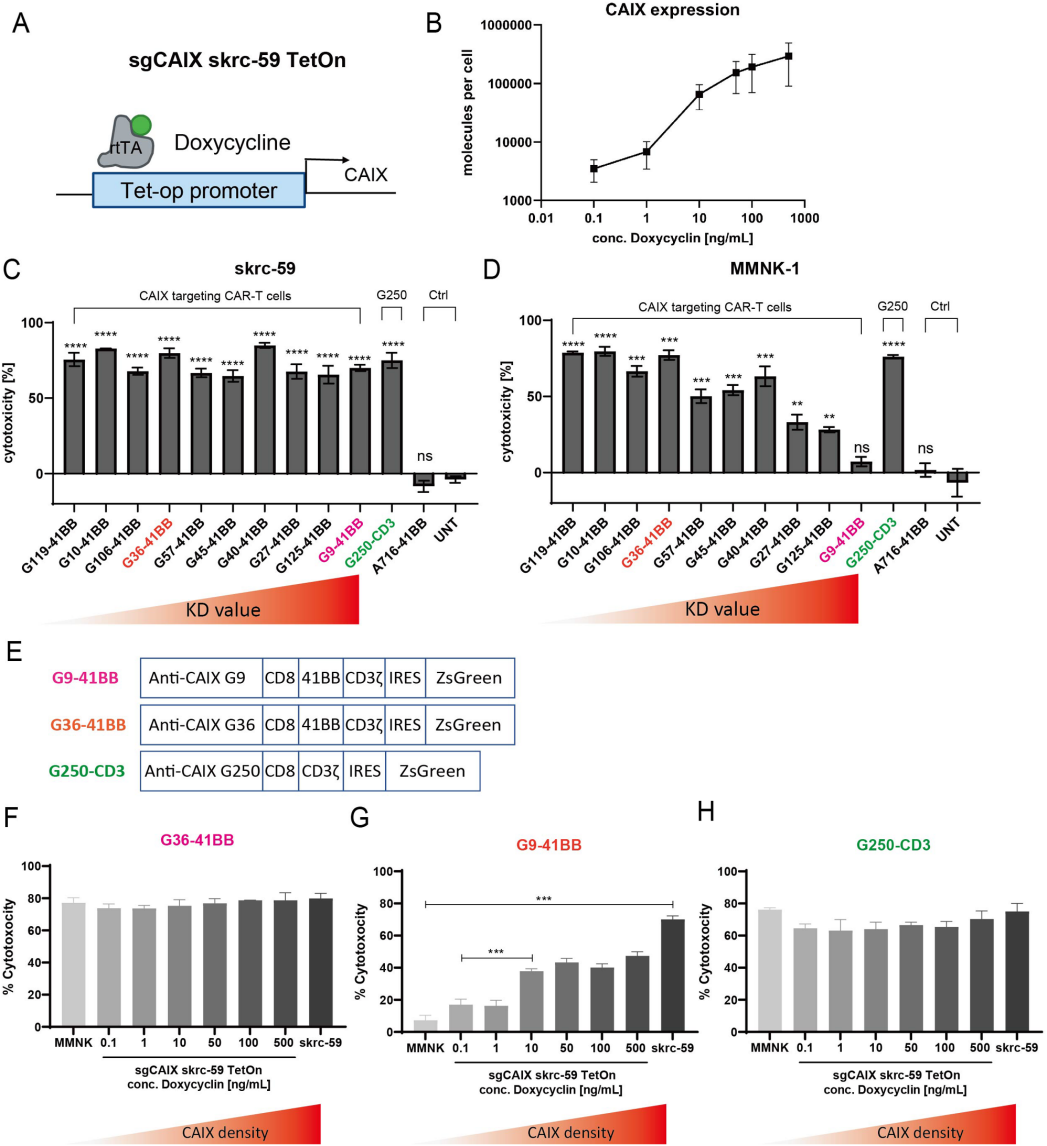
Cytotoxicity of anti-CAIX CAR-T cells in vitro. (**A**) Tet-On CAIX inducible skrc-59 cell line is engineered to utilize the Tet-op promoter to sense different concentrations of Dox to control CAIX expression. (**B**) CAIX expression on the Tet-On cells in the presence of different Dox concentrations. (**C**) Cytotoxicity of anti-CAIX CAR-T cells on CAIX high skrc-59 tumor cells. CD8 CAR-T cells with E:T ratio of 2:1. The variants of CAR-T cells are arranged in descending order of affinity from left to right indicated by increasing KD values. *P* values are defined by unpaired two-tailed t-tests between each CAR-T to untransduced T cell (UNT) (∗*p* < 0.05; ∗∗*p* < 0.01; ∗∗∗*p* < 0.001; and ∗∗∗∗*p* < 0.0001). (**D**) Cytotoxicity of anti-CAIX CAR-T cells on CAIX low MMNK-1 cholangiocytes. CD8 CAR-T cells with E:T ratio of 2:1. From left to right, within the group of CAIX targeted CAR-T cells, the KD value of each scFv is increasing, meaning the affinity is decreasing. *P* values are defined by unpaired two-tailed t-tests between each CAR-T to untransduced T cell (UNT) (∗*p* < 0.05; ∗∗*p* < 0.01; ∗∗∗*p* < 0.001; and ∗∗∗∗*p* < 0.0001). Specificity index is defined by using the cytotoxicity on skrc-59 tumor cells divided by the cytotoxicity on MMNK-1 cells. (**E**) CAR constructs of G9-41BB, G36-41BB and G250-CD3 are shown. Cytotoxicity of (**F**) G36, (**G**) G9 and (**H**) G250 on Tet-On inducible skrc-59 cells. The variants of CAR-T cells are arranged in escalating order of CAIX density on the cell surface. All data with error bars are presented as mean ± SD. *P* values are defined by unpaired two-tailed t-tests (∗*p* < 0.05; ∗∗*p* < 0.01; ∗∗∗*p* < 0.001; and ∗∗∗∗*p* < 0.0001). Only significant differences are shown in the plot

To explore how CAIX antigen density influences CAIX targeted CAR-T efficacy, we transduced sgCAIX skrc-59 cells to express the Tet operator (tetO) followed by human CAIX (termed the Tet-On system) (Fig. 2A). In the presence of doxycycline (Dox), Tet-On skrc-59 cells can be induced to express a wide range of CAIX molecules on the cell surface without interference from the endogenous CAIX gene (Figure S1A). The Tet-On inducible system was quantified using serial Dox concentrations from 0.1 to 500 ng/mL and the results demonstrated that these cells displayed a range of CAIX expression, covering MMNK-1 healthy cells (1,824 CAIX molecules per cell) to skrc-59 tumor cells (123,789 CAIX molecules per cell) and providing an isogenic cell line that can be used for cytotoxicity assessment (Fig. 2B and S1B).

### G9 CAR-T cells maintained the killing on tumor cells and mitigated toxicity on cholangiocytes

We aimed to attenuate the killing of CAR-T cells on CAIX low expressing normal cells to mitigate OTOT of CAIX targeted CAR-T cells. Affinity fine-tuned anti-CAIX CAR-T cells were constructed using a panel of CAIX targeted scFvs obtained from CAIX paramagnetic proteoliposome (PMPL)-based panning against the Mehta I/II non-immune human scFv-phage display libraries [20]. These scFvs have KD (equilibrium dissociation constant) values against CAIX ranging from 1.49 to 99.58 nM, covering two orders of magnitude (Table S1) [20]. As we identified the superior efficacy and persistence of 41BB 2^nd^ generation anti-CAIX CAR (BBζ) in vivo [17], all of the anti-CAIX scFvs were assembled into a BBζ CAR construct. The G250 CAR used in the previous clinical trial [13–15, 23] and comprised of a murine anti-CAIX scFv and a CD3ζ activation domain, was also generated as a control (G250).

To determine the therapeutic index of the CAR-T cells with different scFvs and identify a CAR which can mitigate OTOT, we tested the cytotoxicity of CAR-T cells on CAIX high skrc-59 ccRCC cells and CAIX low MMNK-1 cholangiocytes using an image-based assay developed in our lab [17]. All anti-CAIX CAR-T cells showed significant killing activity against CAIX high skrc-59 cells at an effector to target (E:T) ratio of 2:1 (Fig. 2C), however the cytotoxicity of CAR-T cells on MMNK-1 cells is positively correlated with scFv affinity such that the higher the affinity of the CAR, the stronger the observed killing activity (Fig. 2D). This provided the rationale to fine-tune scFv affinity to mitigate OTOT due to physiological levels of CAIX expression on cholangiocytes. From our panel, G9 showed highly specific killing against CAIX high tumor cells while sparing CAIX low healthy cells with a specificity index of 9.63 compared to G36’s 1.03 and G250’s 0.99 (Fig. 2C-E).

### Killing activity of G9 CAR-T cells is correlated with CAIX density

We further evaluated CAR-T cells, including G9, G36 and G250 on Tet-On sgCAIX skrc-59 cells (Fig. 2F-H and Figure S2). The results showed that G9 CAR-T cells selectively killed tumor cells with high CAIX expression levels and its killing capacity was positively correlated with CAIX expression level on the cell surface (Fig. 2G and S2). Conversely, both G36 and G250 killed even low CAIX expressing cells, providing a possible explanation for the liver toxicity observed in patients treated with G250 CAR-T cells [13–15, 23]. Through examination of the killing specificity of CAR-T cells and their KD, we revealed that the killing specificity is only correlated with KD in the low CAIX expressing cells but not the high CAIX expressing cells (Figure S3).

### G9 scFv-Fc has a low affinity but G9 CAR-T has a high avidity toward skrc-59 tumor cells

**Fig. 3:**
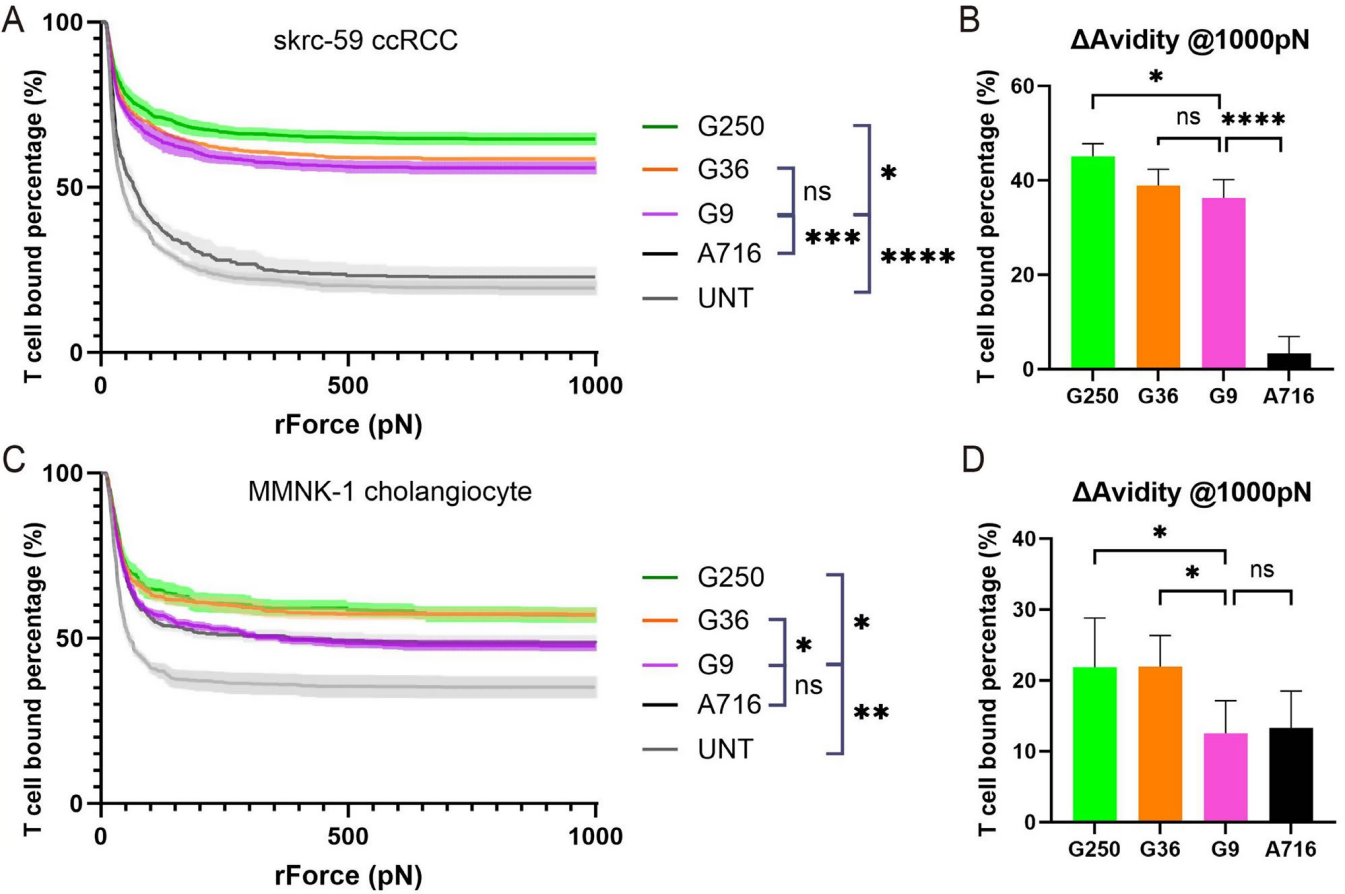
Avidity of CAR-T cells on tumor and normal cells. (**A**) Avidity of CAR-T cells on skrc-59 ccRCC tumor cells. The percentage of G250 (green), G36 (orange), G9 (pink), A716 (black), UNT (grey) binding to skrc-59 ccRCC tumor cells are shown in the plot (n = 4 per group). (**B**) Avidity of CAR-T cells on skrc-59 tumor cells at 1000 pN endpoint. The normalized percentage of G250 (green), G36 (orange), G9 (pink), A716 (black) binding to skrc-59 ccRCC tumor cells at 1000 pN are shown in the bar plot (the normalized percentage of binding is defined by minus the binding of UNT) (n = 4 per group). (**C**) Avidity of CAR-T cells on MMNK-1 cholangiocytes. The percentage of G250 (green), G36 (orange), G9 (pink), A716 (black), UNT (grey) binding to MMNK-1 cholangiocytes are shown in the plot (n = 4 per group). (**D**) Avidity of CAR-T cells on MMNK-1 cholangiocytes at 1000 pN endpoint. The normalized percentage of G250 (green), G36 (orange), G9 (pink), A716 (black) binding to MMNK-1 cholangiocytes at 1000 pN are shown in the bar plot (the normalized percentage of binding is defined by minus the binding of UNT) (n = 4 per group). All data with error bars are presented as mean ± SD. *P* values are defined by unpaired two-tailed t-tests (∗*p* < 0.05; ∗∗*p* < 0.01; ∗∗∗*p* < 0.001; and ∗∗∗∗*p* < 0.0001)

To further investigate the role of cell adhesion in CAR-T and target cell interactions, we used dynamic acoustic force measurements to assess binding avidity. We found that G9 CAR-T cells showed a high binding avidity to skrc-59 cells, clustering with G36 and G250 in the upper panel as compared to control CAR-T anti-B cell maturation antigen (BCMA) A716 and untransduced T cells (UNT). However, G9’s avidity is statistically lower than G250 and also trends lower than G36 (Fig. 3A and B). When we measured the binding avidity of CAR-T cells to MMNK-1 cholangiocytes, we found G9’s avidity was similar to the irrelevant CAR A716 and lower than either G36 or G250 (Fig. 3C and D). These findings are in agreement with the comparable cytotoxicity of G9 to G36 and G250 on skrc-59 tumor cells, and the mitigation of G9 killing of MMNK-1 cholangiocytes (Fig. 2).

### Fine-tuned G9 CAR-T cells showed superior efficacy on primary and lung metastatic ccRCC PDOTS

**Fig. 4:**
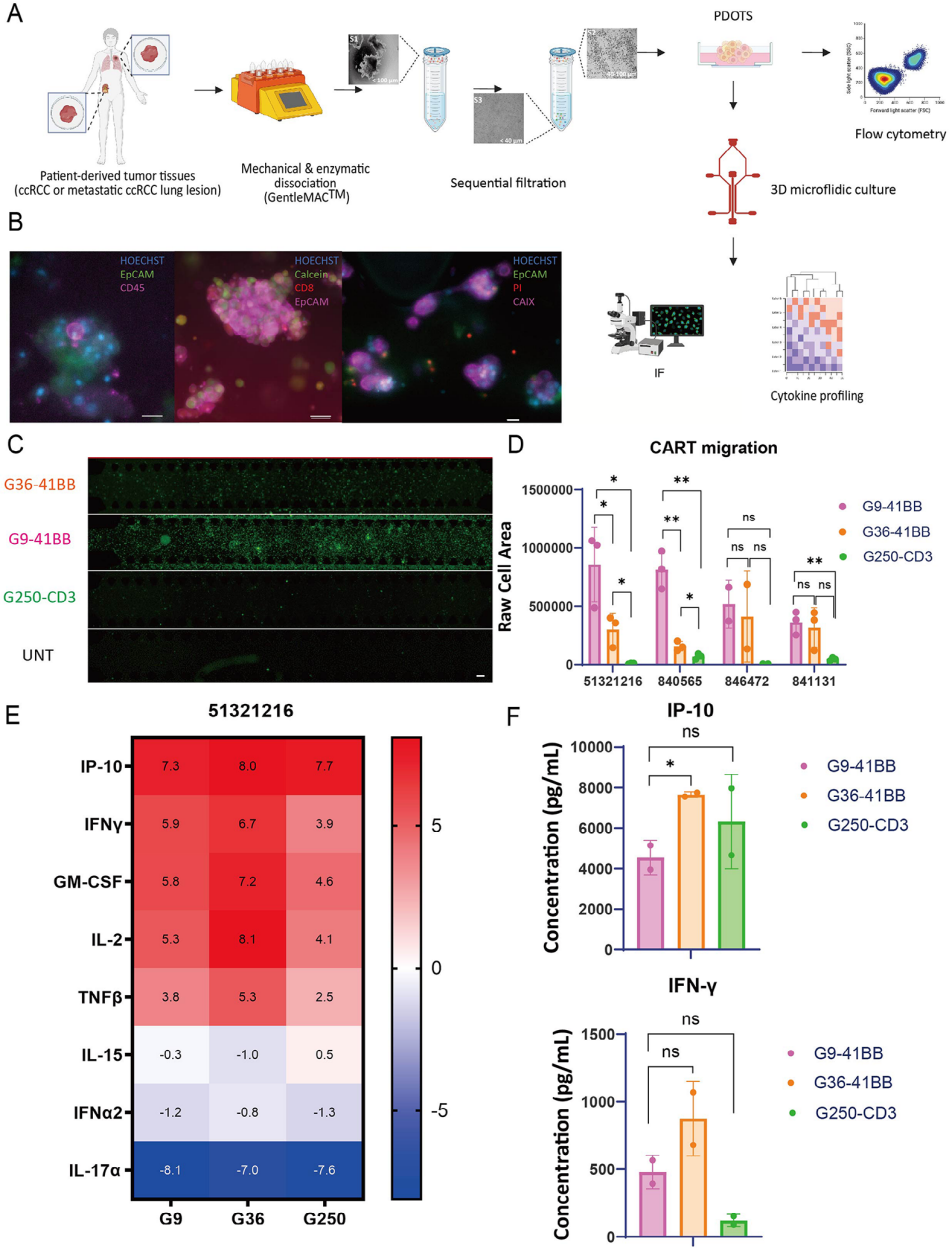
Efficacy of anti-CAIX CAR-T cells on RCC patient derived organotypic tumor spheroids (PDOTS). (**A**) Schematic of workflow. ccRCC patient tumor specimens were collected, digested and filtered into S1, S2, S3 fractions, in which S2 fraction was used to generate ccRCC PDOTS. On Day −1, PDOTS mixed with collagen were injected into the central channel of the microfluidic device. Quality control (QC) was performed on Day 0 to profile ccRCC TME and determine the viability of PDOTS. The CAR-T cells were added to the side channels and co-incubated with PDOTS for 6 days. And the downstream analysis was performed. (**B**) Representative images of PDOTS on Day 0. IF was performed to show biomarkers on PDOTS. In the left panel, PDOTS were stained with Hoechst, EpCAM and CD45. In the middle panel, PDOTS were stained with Hoechst, Calcein, CD8, and EpCAM. In the right panel, PDOTS were stained with Hoechst, EpCAM, PI, and CAIX. Scale bars shown in the images represent 20 μm. (**C**) CAR-T migration was evaluated on Day 6 on PDOTS of a primary ccRCC sample (51321216). By using the ZsGreen fluorescence of CAR-T cells, the signal of middle channel was quantified. Scale bars shown in the images represent 200 μm. (**D**) CAR-T migration was quantified on PDOTS of a primary ccRCC sample (51321216). G9 (pink), G36 (orange), G250 (green) are shown in the plot. (**E**) Heatmap of log2 fold change of selected cytokine and chemokine in the supernatant. Cytokine profiling was performed via Luminex using supernatant collected on Day 6 from PDOTS (51321216 RCC sample) and CAR-T co-cultures. Selected cytokines and chemokines are shown here, including IP-10, IFN-γ, GM-CSF, IL-2, TNFβ, IL-15, IFN-α2, and IL-17α. (**F**) Bar plots of IP-10, and IFN-γ secretion of PDOTS (51,321,216) co-culturing with G9 (pink), G36 (orange), and G250 (green) were shown in the plot. All data with error bars are presented as mean ± SD. *P* values are defined by unpaired two-tailed t-tests (∗*p* < 0.05; ∗∗*p* < 0.01; ∗∗∗*p* < 0.001; and ∗∗∗∗*p* < 0.0001)

Organoid culture systems provide cellular interactions and a complete tumor microenvironment (TME) and have been used widely in tumor immunology and drug testing [24–26]. Patient-derived organotypic tumor spheroids (PDOTS) were generated from primary patient tumor tissue and they were cultured in a custom microfluidic device that allows tumor spheroids to grow in a 3D gel matrix in the center region of the device with media added to the side channels [27, 28]. ccRCC patient tumor specimens were collected and PDOTS were generated using the method described previously [27] (Fig. 4A). The presence of the complex ccRCC TME in the ccRCC PDOTS was confirmed by immunofluorescence (IF). The results showed that ccRCC PDOTS cultures accurately recapitulate the ccRCC TME, including tumor infiltrating lymphocytes (TILs) like CD8 T cells, and CAIX+ tumor cells (Fig. 4B).

The ccRCC PDOTS were generated from four patient samples, including three primary ccRCC tissues (51321216, 840565, 846472) and one lung metastatic lesion (841131). G9, G36 and G250 CAR-T cells (CD4:CD8 = 2:1) were added to the side channel of the microfluidic devices. On Day 6, G9 showed superior migration to the middle channel compared to G36 and G250 CAR-T cells in sample 51321216 and 840565 (Fig. 4C and D). However, we did not observe significant migration of either G9 or G36 compared to G250 in patient 846472 (Fig. 4C and D). By profiling the supernatant of the coculture on Day 6, we identified a significant level of cytokine release from PDOTS treated with G9 and G36, G250 CAR-T cells, including IP-10, IFN-γ, GM-CSF, and IL-2 (Figs. 4E and S4). As can be seen by the overall cytokine profile, PDOTS co-cultured with G9 CAR-T cells secreted a similar level of IP-10 and IFN-γ in comparison to G36 and G250 (Fig. 4F). These results showed that despite a lower affinity, G9 CAR-T cells exhibited similar efficacy compared to G250 and G36 on both primary and metastatic ccRCC PDOTS.

### Fine-tuned G9 CAR-T cells showed superior efficacy compared to G250 in a ccRCC orthotopic mouse model

**Fig. 5:**
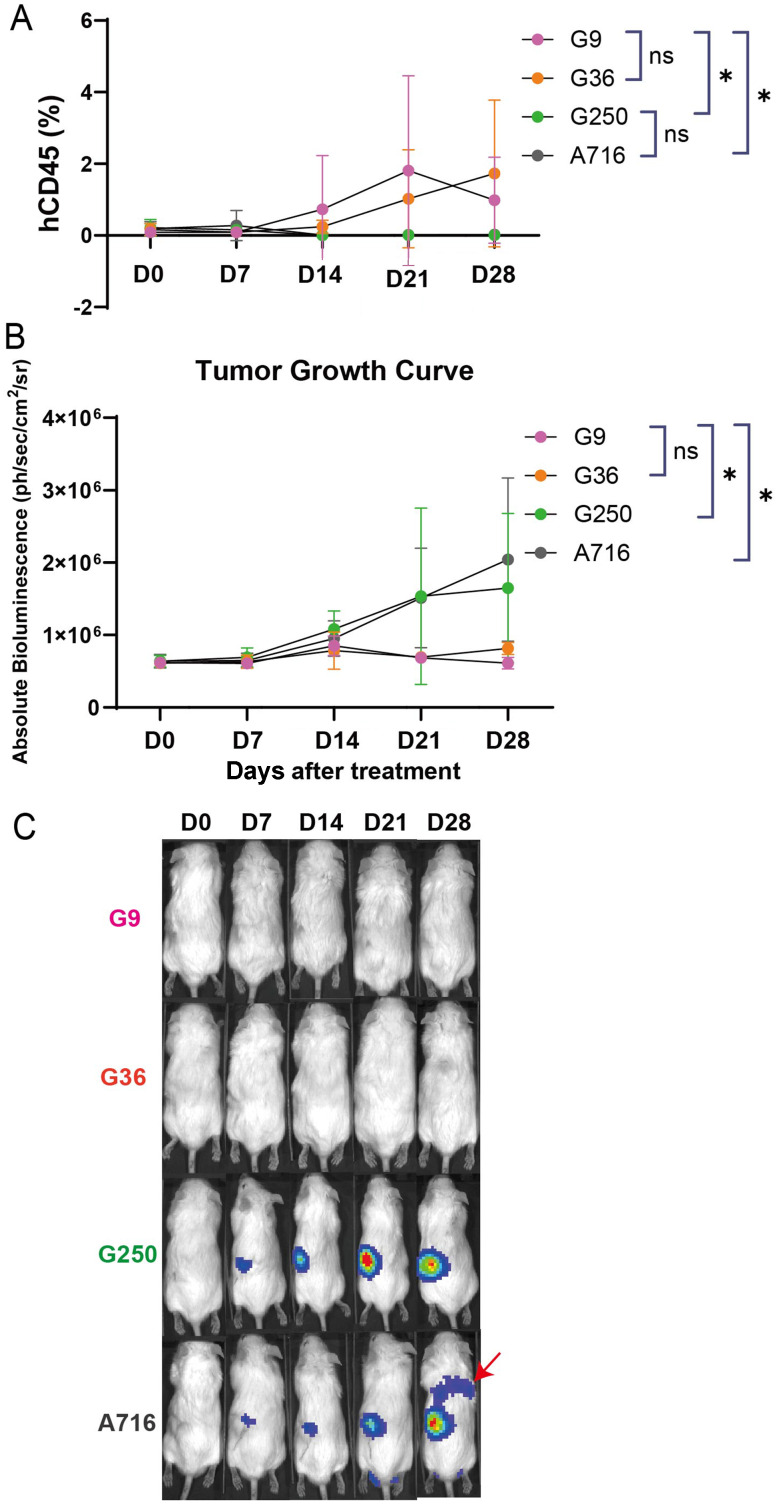
Fine-tuned CAIX targeted G9-41BB CAR-T cells exhibited superior efficacy in a ccRCC orthotopic NSG-SGM3 mouse model. (**A**) CAR-T expansion (the percentage of human CD45 + immune cells out of total live leukocytes in the peripheral blood) of G9 (pink), G36 (orange), G250 (green) and A716 (grey) is shown in the plot (n = 5 per group). Peripheral blood was analyzed via flow cytometry. ccRCC skrc-59 tumor cells were inoculated under the kidney capsule. One week after tumor implantation, one million CAR-T cells were injected intravenously. Tumor growth was monitored by BLI weekly for four weeks and circulating CAR-T cells were phenotyped weekly by flow cytometric analysis of peripheral blood. (**B**) Tumor growth curve of the mice treated with one million CD4:CD8 = 2:1 G9 (pink), G36 (orange), G250 (green) or A716 (grey) CAR-T cells (n = 5 per group). BLI was performed on Day 0, Day 7, Day 14, Day 21 and Day 28 after CAR-T infusion. (**C**) BLI images of mice treated with one million CD4:CD8 = 2:1 G9 (pink), G36 (orange), G250 (green) or A716 (grey) CAR-T cells on Day 0, Day 7, Day 14, Day 21 and Day 28 after CAR-T infusion. The red arrow indicates the lung metastasis of RCC tumor. All data with error bars are presented as mean ± SD. *P* values are defined by unpaired two-tailed t-tests (∗*p* < 0.05; ∗∗*p* < 0.01; ∗∗∗*p* < 0.001; and ∗∗∗∗*p* < 0.0001)

To assess the ability of CAR-T cells to control tumor growth in vivo, we established a ccRCC orthotopic tumor bearing NSG-SGM3 mouse model where luciferized CAIX+ human ccRCC skrc-59 tumor cells are implanted under the murine kidney capsule [17]. One week after implantation, tumor engraftment was confirmed by bioluminescence imaging (BLI), followed by randomized grouping of the mice (n = 5 per group). Four groups were tested in this model, including G250, G36, G9 and A716 at a single dose of one million CAR-T cells with a CD4:CD8 T cell ratio of 2:1 (Figs. 5 and S5). Tumor growth and CAR-T cell expansion and persistence were evaluated weekly via BLI and flow cytometric analysis of peripheral blood respectively.

G9 CAR-T cells showed superior expansion in vivo compared to the G250 CAR-T and similar expansion compared to G36 (Fig. 5A). In addition, during the 4 weeks post injection, G9 treatment resulted in significant antitumor activity comparable to G36 while G250 was not able to control tumor growth (Figs. 5B and C, S1 and S6). However, no significant body weight loss (Figure S7) or histopathological changes in bile duct were observed in the mice treated with G9 CAR-T cells (Figure S8).

### G9 recognizes a different epitope of CAIX compared to G250

**Fig. 6:**
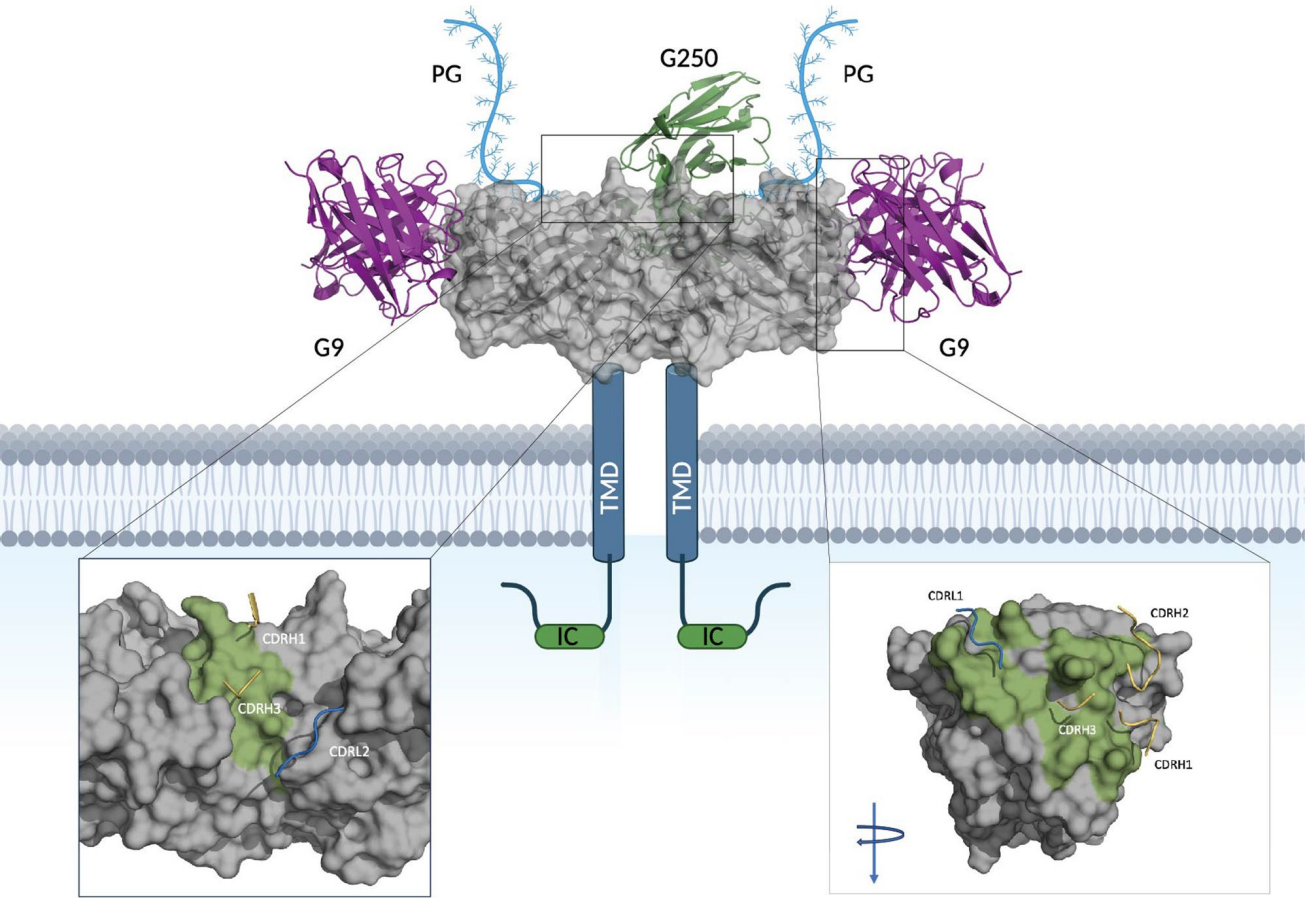
Binding mode of G9 and G250 with CAIX. Depicts TAA, CAIX, gray, along with its transmembrane domain, intracellular tail and proteoglycan (PG) domain at the N-terminus. The variable regions of mAb, G9, are shown in pink bound to the predicted epitope and the variable regions of mAb G250, are shown in green. The zoomed view depicts the binding interface of G250 to CAIX (left) and G9 to CAIX (right, turned 90 degree) with epitopes colored in green and contact regions of the complementarity determining regions (CDRs) shown in yellow (heavy chain), and blue (light chains). G9 shows 2:1 binding mode to a CAIX dimer and G250 binds to a CAIX dimer in 1:1 ratio

Through subunit-based epitope mapping, Xu et al. found that the G9 scFv-Fc binds to both full length CAIX and the catalytic core, however it does not bind to the proteoglycan (PG) domain or inhibit catalytic activity of CAIX, indicating that it targets an epitope distal to the catalytic domain [20]. Through *in silico* computational docking, and in agreement with these findings, we found that G9 likely interacts with CAIX through helices on the periphery of CAIX in a manner that would not inhibit the catalytic activity of CAIX (Fig. 6). The key hydrogen bond interactions to CAIX are maintained by CDRH1 (T28, S31, and Y32), CDRH2 (S51, S53, and G55 (backbone)), CDRH3 (S95 and S97) and CDRL1 (R29, G30 (backbone), and N32). In addition, pi-alkyl interactions are formed between Y31 and E169 in CDRL1 and CAIX, respectively. G9 contacts alpha helices distal to the catalytic site in the following regions, ENSAYE and SPLEEIAEE. This binding mode is in contrast to that of G250 mAb which is modeled to bind to the interface of two CAIX monomers through interactions with the following linear motifs, ALGPGREYRAL and LSTAFARV, the same as reported previously [29].

## Discussion

The success of CAR-T cell therapies for the treatment of hematologic malignancies has not only been due to their potency in killing tumor cells but also because of the exquisite nature of the B lineage specific CD19, CD20 and BCMA markers to which their targeting moieties are directed [30–37]. In these disease settings, the use of high-affinity CAR-T cells directed to TAAs holds an advantage that is generally not true for solid tumors where solid tumor related TAAs are expressed in the normal tissues [38]. In this study, we have successfully designed TAA CAIX targeted therapy through fine-tuning the affinity/avidity of the anti-CAIX scFv. Anti-CAIX G9 CAR-T cells exhibited superior antitumor efficacy on the CAIX high expressing tumor cells and mitigated OTOT toxicity on normal tissues with CAIX low expression.

The carbonic anhydrase (CA) family consists of 15 isoforms of ubiquitous metalloenzymes that catalyze the reversible hydration of carbon dioxide to bicarbonate and protons [39, 40]. CAIX is a zinc enzyme CA which binds to anion exchanger 2 (AE2) receptor, increasing bicarbonate transport and maximizing acid secretion [39]. CAIX is distinguished from its other family members due to its unique evolutionary feature: an N-terminus PG domain, which also contributes to a better catalytic activity for carbon dioxide hydration at more acidic pH values [41, 42]. We hypothesize that the interface epitope G250 recognizes would result in a 1:1 stoichiometric ratio of G250:CAIX dimer due to steric clashing of a second G250 scFv. However, G9 would be able to bind the CAIX dimer in a 2:1 mode, leading to a higher avidity. In addition, the G9 epitope is membrane proximal compared to G250’s. CAR-T cells that bind to membrane proximal epitopes have demonstrated different therapeutic effects compared to the ones that target membrane distal epitopes [43]. As such, the CAIX membrane proximal epitope targeted by G9 should be used for other CAIX directed therapies as it can lead to greater cytotoxic activity.

CAIX is regulated by the Von Hippel Lindau (*VHL*) protein (pVHL) [44] and serves as a hallmark for multiple solid tumors [45], including RCC [46], HNSCC [47], glioblastoma [48], breast cancer [49], mesothelioma [50], and bladder cancer [51]. CAIX exerts its physiological functions even at the acidic pH typical in the TME of solid hypoxic tumors (pH = ~ 6.5), leading to the resistance to conventional chemo- and radiotherapy [41]. High CAIX expression is associated with early disease stage and grade [52], and negatively correlated with patient prognosis [53]. PDOTS generated from lung metastatic RCC responded well towards G9 CAR-T cell therapy, which might result from a similar CAIX expression in metastatic lung lesions compared to the primary RCC.

Low affinity/avidity CAR-T cell therapy has shown enhanced cytotoxicity [54–56], elevated expansion [55, 57], decreased exhaustion [58], better migration and trafficking [56], as well as increased selectivity [54]. In our study comparing G9 to G250, we observed superior in vivo antitumor efficacy as well as boosted in vivo expansion, better ex vivo migration and a wider therapeutic window of G9. To further enhance tumor killing efficacy and decrease the OTOT of CAIX targeted CAR-T cells, we have designed an anti-CAIX/CD70 dual-targeted CAR-T cell therapy that is activated in the presence of either CAIX or CD70, thus killing CAIX low expressing tumor cells and preventing potential antigen escape [59].

In skrc-59 tumor bearing mice treated with G9 CAR-T cells, no significant body weight loss or histopathological changes in the bile duct were observed (Figures S7 and S8). However, this model has its limitation due to the expression of murine CAIX instead of human CAIX in the mouse bile duct. To assess in vivo OTOT toxicity, we investigated MMNK-1 tumorgenesis. Unfortunately, MMNK-1 cells are not tumorigenic in immunodeficient mice [22]. Castellarin et al. reported a mouse model with human HER2 expression on mouse normal hepatocytes to access OTOT toxicity of HER2 targeted CAR-T cells [56]. By transducing adeno-associated virus serotype 8 (AAV8) encoding the human CAIX gene and a fluorescent reporter into NSG-SGM3 mice, we anticipate the development of a model that would serve as a useful tool to evaluate OTOT of anti-CAIX CAR-T cells. Moreover, the Tet-On system we established could also be applied to the mouse model to recapitulate physiological CAIX expression by feeding the mice with Dox containing water [60].

In addition to OTOT toxicities, the two main toxicities resulting from CAR-T cell infusions are cytokine release syndrome (CRS) and immune effector cell-associated neurotoxicity syndrome (ICANS), and they are often not revealed in mouse models but are eventually discovered in clinical trials [61]. The affinity and avidity of the CAR moieties towards their target antigen not only affect tumor cell killing, but also CAR-T cell cytokine release [54, 62]. Using PDOTS 3D ex vivo cultures, we were able to profile the cytokine release in the cocultures, making PDOTS a potential tool to study CRS of CAR-T therapy ex vivo.

Potential limitations of our study include the lack of validation of G9 CAR-T in vivo efficacy using other models than the skrc-59 tumor bearing NSG-SGM3 mouse. Further evaluation of CRS and ICANS can be performed on a humanized mouse model [63, 64]. Moreover, crystallization or cryogenic electron microscopy (Cryo-EM) can be utilized to validate the binding mode of G9 and G250 and to study their CAIX epitopes and accessibility.

## Materials and methods

### Cell lines and culture

Human clear cell renal cell carcinoma cell line skrc-59 (obtained from Dr. Gerd Ritter, Memorial Sloan-Kettering Cancer Center, New York, USA) was engineered to express high levels of human CAIX (skrc-59 CAIX+) [21] and mCardinal fluorescent protein. Using CRISPR Cas9, sgCAIX skrc-59 cell line was engineered to knock out CAIX. MMNK-1 cholangiocyte cell line [22] was purchased from Japanese Collection of Research Biosources Cell Bank (JCRB). These cells were grown in RPMI-1640 or DMEM medium (Gibco) supplemented with 10% (v/v) heat-inactivated fetal bovine serum (FBS, Gibco) at 37 °C with 5% CO_2_.

### Production of lentivirus particles

For lentivirus production, polyethylenimine (PEI), DNA of the helper plasmids VSVG, TAT, GAG and REV (10 µg per 15 cm dish of 293T cells) and 20 µg of the respective CAR DNA were added to Opti-MEM medium (Gibco). This mixture was incubated for 20 min at RT and was afterwards added drop by drop to a 15 cm dish of LentiX-293T cells (Clontech). After 48 h of incubation, the supernatant was collected, debris was removed and lentiviral concentrator (Clontech) was added in a 1:3 (v/v) ratio. This mixture was incubated overnight at 4 °C. The next day the tubes were centrifuged for 45 min at 1,500 g and the supernatant was discarded. The pelleted lentivirus was resuspended in RPMI-1640 medium and stored at -80 °C.

### Establishment of Tet-on skrc-59 cell line

Tet-On vector (obtained from Dr. Ming-Ru Wu, Dana-Farber Cancer Institute, MA, USA) was packaged into lentivirus and transduced into sgCAIX skrc-59 cells. In the presence of doxycycline, the skrc-59 CAIX+ cells were sorted as CAIX Tet-On skrc-59 cells using SONY sorter MA900.

### Generation of CAR-T cells

Apheresis leukoreduction collars were obtained from the blood bank of the Brigham and Women´s Hospital under DFCI approved IRB protocol #14-343. Human peripheral blood mononuclear cells (PBMCs) were separated using Ficoll-Paque-PLUS (GE Healthcare). CD4 and CD8 T cells were isolated by positive selection using CD4 MicroBeads and CD8 MicroBeads (Miltenyi Biotec) respectively. T cells were cultured in RPMI-1640 medium supplemented with 10% FBS, IL-21 (30 ng/mL, Miltenyi Biotec) and activated by T cell TransAct (Miltenyi Biotec). CAR-T cells were generated by lentiviral transduction (MOI=20) with 10 µg/mL Diethylaminoethyl (DEAE). The CAR positive T cells were sorted by SONY sorter MA900. After sorting, the T cells were cultured in RPMI-1640 medium supplemented with 10% FBS, IL-7 (500 IU/mL, Miltenyi Biotec), IL-15 (84 IU/mL, Miltenyi Biotec) and Gentamicin (50 µg/mL) (Gibco).

### Immunohistochemical staining

IHC staining was performed on formalin-fixed, paraffin-embedded (FFPE) 4 μm tissue sections. An in-house IHC assay was used to optimize CAIX (1:40,000, MN-75, mouse monoclonal antibody [65]). Tissue slides were baked at 60 °C for 30 min and then stained on BOND III Autostainer (Leica Biosystems) using BOND Polymer Refine Detection Kit (DS9800, Leica Biosystems). Antigen retrieval was performed with BOND Epitope Retrieval Solution 1 (Citrate pH = 6, Leica Biosystems) for 20 min. Slides were counterstained with hematoxylin and dehydrated in graded ethanol solutions and xylene prior to mounting and coverslipping.

### IHC image analysis

The immunostained slides were scanned at 20X magnification using Aperio ScanScope (Leica Microsystems). For each slide, viable tumor areas were manually annotated using the HALO software. Then, classifiers were created to accurately identify tumor cells within the annotated areas by excluding stroma, immune cells and red blood cells. The number of positive CAIX positive tumor cells was determined using the HALO platform multiplex-IHC algorithm, version 2.1.1637.18 (Indica lab). Image analysis results were then validated through visual inspection by pathologists with expertise in the evaluation of IHC stains in RCC (M. Ficial, S. Signoretti).

### Quantification of CAIX via QuantiBrite beads

Skrc-59 Tet-On CAIX cells were counted and seeded at a concentration of 3*10^5^ cells/mL into 6-well plates. The respective doxycycline concentration (0.1–1,000 ng/mL) was added and at the indicated time points and the samples were collected by detaching the cells with trypsin (Corning). The samples were incubated with human Fc blocking solution (1:500) for 10 min at RT, then washed and stained with anti-CAIX-PE antibody (clone REA658, Miltenyi Biotec) for 15 min at RT. After three washing steps, the samples were fixed with 2% paraformaldehyde (PFA) for 15 min at RT and were analyzed together with QuantiBrite PE beads (Becton Dickinson) using flow cytometry (LSRFortessa, BD Biosciences). Data was analyzed using FlowJo software (FlowJo LLC).

### Quantification of CAIX via dSTORM

Fresh frozen human tissue samples were collected under DFCI approved protocols #01-130 and #19-194. The samples were sectioned using a cryo-microtome. 10 μm thick sections were mounted on poly-L-lysine (Sigma-Aldrich) coated high precision coverslips (#1.5 H, Marienfeld) and air dried for 10 min before storage at -80^o^C. Before staining, samples were rehydrated using phosphate buffered saline (pH 7.4, Gibco) then fixed with 4% paraformaldehyde (PFA) in PBS (pH 7.4, Sigma-Aldrich) for 10 min. Samples were washed and free aldehydes were quenched using Tris buffered saline (Sigma-Aldrich).

Fixed sections were blocked and permeabilized using a solution containing 0.3% Triton X-100 (Sigma-Aldrich), 10% normal donkey serum (Sigma-Aldrich) and 2% bovine serum albumin (Sigma-Aldrich) for 2 h at room temperature. Dylight 550 conjugated primary antibodies against CAIX (NB100-417R, Novus) were diluted in PBS containing 0.3% Triton X-100, 1% normal donkey serum and 0.2% bovine serum albumin and incubated for two hours at room temperature in a humidifying chamber. Subsequently, samples were washed with PBS before applying Bcubed (Oxford NanoImaging) imaging buffer.

Single molecule data acquisition was carried out on the Nanoimager S running NimOS version 1.4 (Oxford NanoImaging). The images were acquired using a 100 × 1.4 NA Olympus objective, sCMOS camera (Hamamatsu orca flash 4.0 V3). 10,000 frames were recorded for the detection of Dylight550 signal with the 561 nm laser in total internal reflection fluorescence illumination (TIRF) mode. Drift correction, localization filtering and data analysis were performed in ONI’s cloud-based data analysis platform (CODI). Photon count, localization precision and sigma value filters were applied to reduce background localizations. CAIX clusters were detected and quantified using a hierarchical density based clustering algorithm (HDBSCAN). Cluster density per area (µm^2^) was used to represent CAIX expression in human tissue samples.

### In vitro killing assay

Celigo in vitro killing assay was performed as described previously [17, 19, 66]. Approximately 3,000 mCardinal + skrc-59 tumor cells or MMNK-1 cholangiocytes or Tet-On skrc-59 cells in the presence of different concentrations of Dox (target cells) were seeded in a 96-well plate (Greiner 655090). After 12 h of incubation, the plate was scanned and analyzed in bright field and far-red channel for mCardinal at the 0 h time point. CAR-T cells were added and co-incubated with the target cells. Additional control wells were prepared with target cells only (negative control) and target cells with 1% Triton-X (positive control). Subsequently, the plate was scanned and analyzed at the 48 h time point with the equation,

$${\text{Cytotoxicity}}\,\% \, = \,\frac{{{\text{negative}}\,{\text{control}}\, - \,{\text{treatment}}}}{{{\text{negative}}\,{\text{control}}\, - \,{\text{positive}}\,{\text{control}}}}\, \times \,100$$

### Cell binding avidity assay

Single-cell suspensions of skrc-59 or MMNK-1 cells at 5*10^7^/mL were prepared by treating the cultured cells with TrypLE (Thermo Scientific, 12604013) for 5 min at 37^o^C. The suspended cells were added to the z-Movi (LUMICKS) microfluidic chips coated with either poly-L-lysine or Concanavalin A for at least 2 h of attachment. Thawed and overnight rested T cells were stained with Celltrace Far Red Cell Proliferation Kit (Thermo Scientific, C34564) according to the manufacturer’s protocol prior to the avidity assay. For each experiment, 5*10^6^/mL CAR-T cells were introduced into the microfluidic chip and incubated for 2.5-5 min. After the incubation, an acoustic force ramp was applied to detach the T cells on the z-Movi system. The order of effector cell addition was randomized between different chips. Data analysis was performed using Oceon software 1.2.8 and statistics were assessed by Prism GraphPad 9.4.1.

### Patient-derived organotypic tumor spheroids (PDOTS)

Fresh human tissue samples were collected under DFCI approved protocols #01-130 and #19-194. Renal tumor samples were dissociated by a semi-automated combined mechanical/enzymatic process. The fresh tumor tissue was cut into pieces of 2–3 mm in size and transferred to C Tubes (Miltenyi Biotech) containing a mix of Enzymes H, R and A (Tumor Dissociation Kit, human; Miltenyi Biotech). Mechanical dissociation was accomplished by performing a program (37C_h_TDK_1 for primary RCC and 37C_h_TDK_2 for lung metastatic RCC) on the gentleMACS Octo Dissociator with Heaters (Miltenyi Biotech). Dissociated material was strained over 100-mm filter and 40-mm filters to generate S1 (> 100 mm), S2 (40–100 mm), and S3 (< 40 mm) spheroid fractions, which were subsequently maintained in ultralow-attachment (ULA) tissue culture plates (Corning). S2 fractions were used for ex vivo culture by resuspending them in type I rat tail collagen (Corning, 354236) at a concentration of 2.8 mg/mL prior to loading into the center gel region of the three-dimensional (3D) microfluidic culture device (AIM Biotech, DAX-1) and incubation for 40 min at 37 ^o^C in humidity chambers to allow for polymerization. Collagen hydrogels containing PDOTS were hydrated with media.

### Immunofluorescence (IF) and migration quantification of PDOTS

Calcein, propidium iodide staining was performed (Nexcelom, CS2-0106) using 1 µM of Calcein (Thermo Fisher Scientific, C34858) and 1 mg/mL of PI (Thermo Fisher Scientific, P3566). Following incubation with the dyes (20 min at room temperature in the dark), PDOTSs were washed with PBS and blocked with FcR blocking reagent (Miltenyi Biotec, 130-059-901) for 30 min at room temperature. Directly conjugated antibodies CD326 EpCAM-FITC (clone 9C4, BioLegend), CD326 EpCAM-AlexaFluor647 (clone 9C4, BioLegend), CD45-AlexaFluor647 (HI30, BioLegend), CD8-PE (clone HIT8a, BioLegend), and CAIX-APC (clone REA658, Miltenyi Biotec) were diluted in 10 mg/mL solution of Hoechst 33342 in PBS and loaded into microfluidic devices for 1 hour incubation at room temperature in the dark. Spheroids were washed twice with PBS with 0.1% Tween20 followed by PBS. Images were captured using 4 objective of a Nikon Eclipse 80i fluorescence microscope equipped with automated motorized stage (Proscan), Z-stack (Prior), and Zyla 5.5 sCMOS camera (Andor). Image analysis was performed using NIS-Elements AR software package version 5.00.00 64-bit. For migration quantification, after 6-day co-incubation of CAR-T and PDOTS, the images were captured as mentioned above and quantitation was performed by measuring total cell area of FITC channel as CAR-T cells express ZsGreen fluorophore.

### In vivo orthotopic humanized ccRCC model

The experiment was performed under DFCI approved protocol #05-035. 50,000 skrc-59 CAIX+ luciferase+ cells were resuspended in 10 µL of RPMI-1640 medium and diluted 1:1 in Matrigel (Corning). This cell mixture was injected under the left kidney capsule of NSG-SGM3 mice (Jackson Laboratories). One week after, tumor engraftment was confirmed with bioluminescence (BLI) imaging and 1 million CAR-T cells were injected through the tail vein of the mice (Day 0, *n*=5 mice per group). The tumor BLI was performed weekly for 4 weeks post CAR-T cell injection. On Day 28, the mice were sacrificed by CO_2_ inhalation, and final blood was drawn. Tissues were collected for H&E staining.

### Flow cytometric analysis of mouse peripheral blood

CAR-T expansion was evaluated using Zombie Yellow™ Fixable Viability Kit (BioLegend), human CD45 (clone HI30, BioLegend), and mouse CD45 (clone 30F11, BioLegend). All samples were analyzed with an LSR Fortessa (BD Bioscience) and data was analyzed using FlowJo software (FlowJo LLC).

### Bioluminescence imaging (BLI)

Tumor growth was monitored weekly using the IVIS Spectrum In Vivo Imaging System (PerkinElmer). Briefly, mice were injected subcutaneously with 75 mg/kg D-luciferin potassium salt (Promega, E1605) in sterile PBS and anesthetized with 2% isoflurane in medical air. Serial bioluminescence images were acquired using the automated exposure set-up. The peak bioluminescence signal intensity within selected regions of interest (ROI) was quantified using the Living Image Software (PerkinElmer), and expressed as photon flux (p/sec/cm^2^/sr). Representative planar bioluminescence images were displayed with indicated adjusted minimal and maximal thresholds.

### In silico docking

The paratope of mAb, G9, was predicted using the ProABC-2 webserver [67, 68]. The structure of CAIX used in docking studies was modeled using the crystal structure (6FE2) [69] as a template through the Alphafold2 Google Collaboratory Notebook [70]. The variable regions of G9 were modeled using the DeepAb notebook [71]. Docking was performed using the ClusPro Webserver [72–76] with an attractive force set to the predicted paratope from ProABC-2. To determine overall contribution of individual residues molecular dynamics simulations (MM/GBSA) were performed through the Hawkdock Webserver [77] on selected models. The highest ranked model by free energy was rendered using the pymol molecular visualization suite. Additional image processing was completed using BioRender.

### Electronic supplementary material

Below is the link to the electronic supplementary material.

**Supplementary Material 1: Supplementary Figure 1.** Quantification of CAIX expression on Tet-On inducible CAIX expressing skrc-59 cells comparing to MMNK-1 and skrc-59 cells. (**A**) Quantification of CAIX expression on Tet-On inducible CAIX expressing skrc-59 cells treated with 100 ng/mL Dox for 120 hours in total. Quantification was performed on 19 time points, 0, 6, 12, 18, 24, 30, 36, 42, 48, 54, 60, 66, 72, 78, 84, 90, 96, 108, 120h. Two arrows indicate the time points of 48 and 96h when the culture media was replaced with fresh one considering the half-life of Dox. (**B**) CAIX quantification results of skrc-59, MMNK-1, and Tet-On inducible CAIX expressing system were shown in the table

## Abbreviations

CAR: chimeric antigen receptor
TSAs: tumor-specific antigens
TAAs: tumor-associated antigens
EGFR: epidermal growth factor receptor
HER2: human epidermal growth factor receptor 2
MUC1: mucin 1
CEA: carcinoembryonic antigen
OTOT: on-target off-tumor toxicity
ccRCC: clear cell renal cell carcinoma
CAIX: carbonic anhydrase IX
HIF: hypoxia inducible factor
scFv: single-chain variable fragment
FFPE: formalin-fixed paraffin-embedded
dSTORM: direct stochastic optical reconstruction microscopy
tetO: Tet operator
Dox: doxycycline
PMPL: paramagnetic proteoliposome
E:T: effector to target
BCMA: B cell maturation antigen
UNT: untransduced T cells
TILs: tumor infiltrating lymphocytes
TME: tumor microenvironment
PDOTS: patient-derived organotypic tumor spheroids
IF: immunofluorescence
CRS: cytokine release syndrome
BLI: bioluminescence imaging
PG: proteoglycan
RCC: renal cell carcinoma
CA: carbonic anhydrases
pVHL: von Hippel-Lindau protein
HNSCC: head and neck single cell carcinoma
KD: dissociation constant
PEI: polyethyleneimine
IHC: Immunohistochemistry
Cryo-EM: cryogenic electronic microscopy
DEAE: diethylethanolamine
mAb: monoclonal antibody
sgRNA: single guided RNA
AAV8: adeno-associated virus serotype 8
CDRs: complementarity determining regions
ICANS: immune effector cell-associated neurotoxicity syndrome
