# Supplementary material for: Affinity fine-tuning anti-CAIX CAR-T cells mitigate on-target off-tumor side effects
Source: Mol Cancer. 2024 Mar 16;23:56. doi: 10.1186/s12943-024-01952-w (PMC10943873; doi:10.1186/s12943-024-01952-w)
Supplement: Supplementary file 1 — Supplementary Material 1: Supplementary Figure 1. Quantification of CAIX expression on Tet-On inducible CAIX expressing skrc-59 cells comparing to MMNK-1 and skrc-59 cells. (A) Quantification of CAIX expression on Tet-On inducible CAIX expressing skrc-59 cells treated with 100 ng/mL Dox for 120 hours in total. Quantification was performed on 19 time points, 0, 6, 12, 18, 24, 30, 36, 42, 48, 54, 60, 66, 72, 78, 84, 90, 96, 108, 120h. Two arrows indicate the time points of 48 and 96h when the culture media was replaced with fresh one considering the half-life of Dox. (B) CAIX quantification results of skrc-59, MMNK-1, and Tet-On inducible CAIX expressing system were shown in the table [file 12943_2024_1952_MOESM1_ESM.docx]

**Supplementary Materials**

**Supplementary Figures**

**
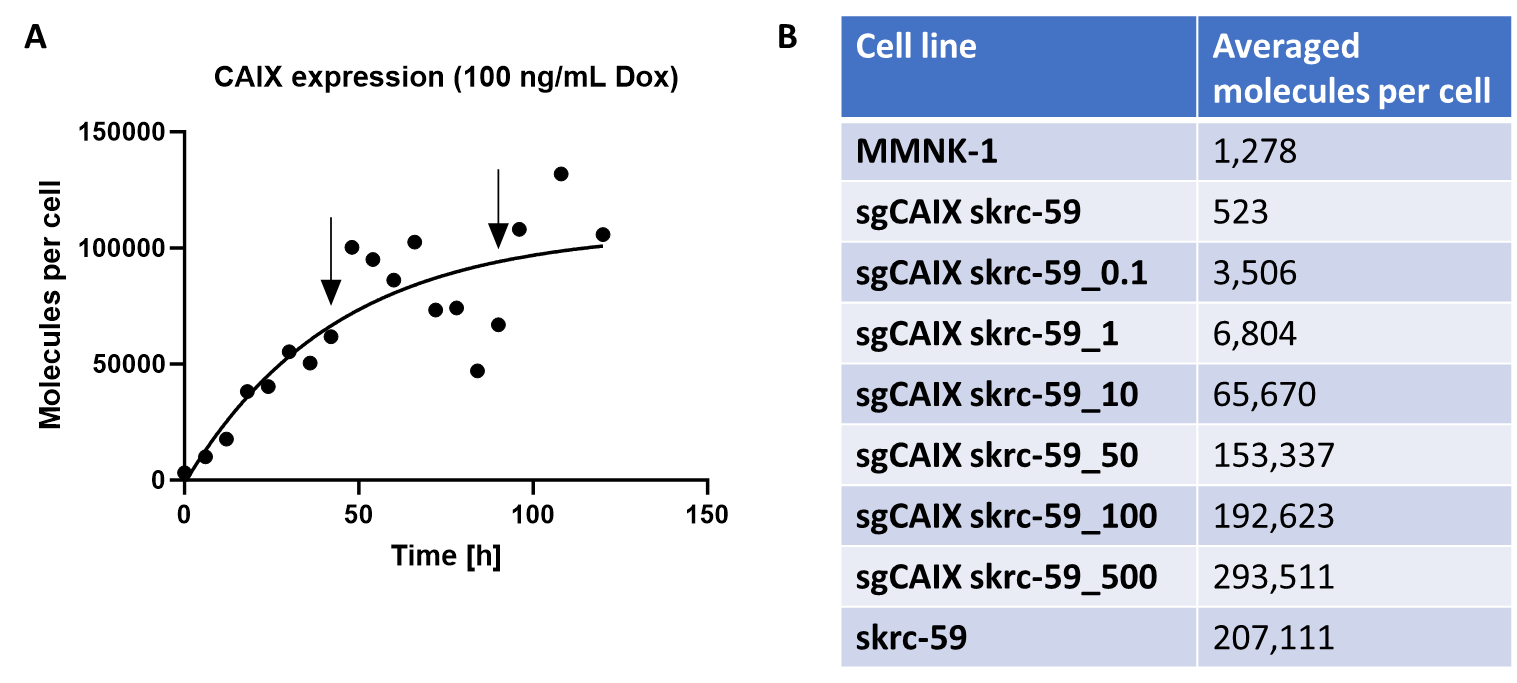
**

**Supplementary Figure 1. Quantification of CAIX expression on Tet-On inducible CAIX expressing skrc-59 cells comparing to MMNK-1 and skrc-59 cells.** **(A)** Quantification of CAIX expression on Tet-On inducible CAIX expressing skrc-59 cells treated with 100 ng/mL Dox for 120 hours in total. Quantification was performed on 19 time points, 0, 6, 12, 18, 24, 30, 36, 42, 48, 54, 60, 66, 72, 78, 84, 90, 96, 108, 120h. Two arrows indicate the time points of 48 and 96h when the culture media was replaced with fresh one considering the half-life of Dox. **(B)** CAIX quantification results of skrc-59, MMNK-1, and Tet-On inducible CAIX expressing system were shown in the table.


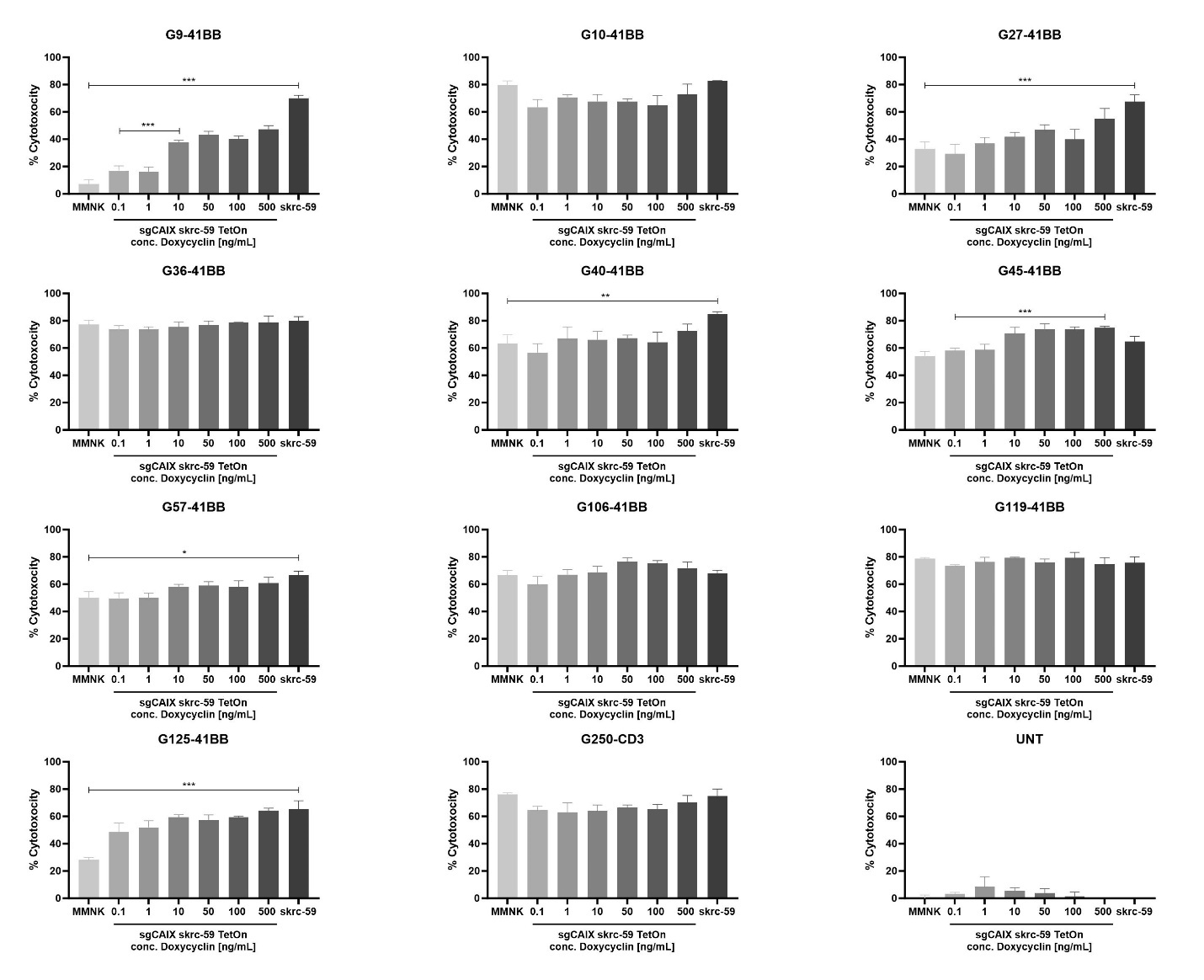


**Supplementary Figure 2. Cytotoxicity of fine-tuned CAR-T cells.** Cytotoxicity of CD8 CAR-T cells in an effector to target cell (E:T) ratio of 2:1 towards skrc-59 CAIX+ tumor cells, MMNK-1 cells, and skrc-59 Tet-On CAIX cells in the presence of different Dox concentrations (0.1 – 500 ng/mL) after treatment for 48 h. All data with error bars are presented as mean ± SD. *P* values are defined by unpaired two-tailed t-tests (∗p < 0.05; ∗∗p < 0.01; ∗∗∗p < 0.001; and ∗∗∗∗p < 0.0001). Only significant differences are shown in the plots.


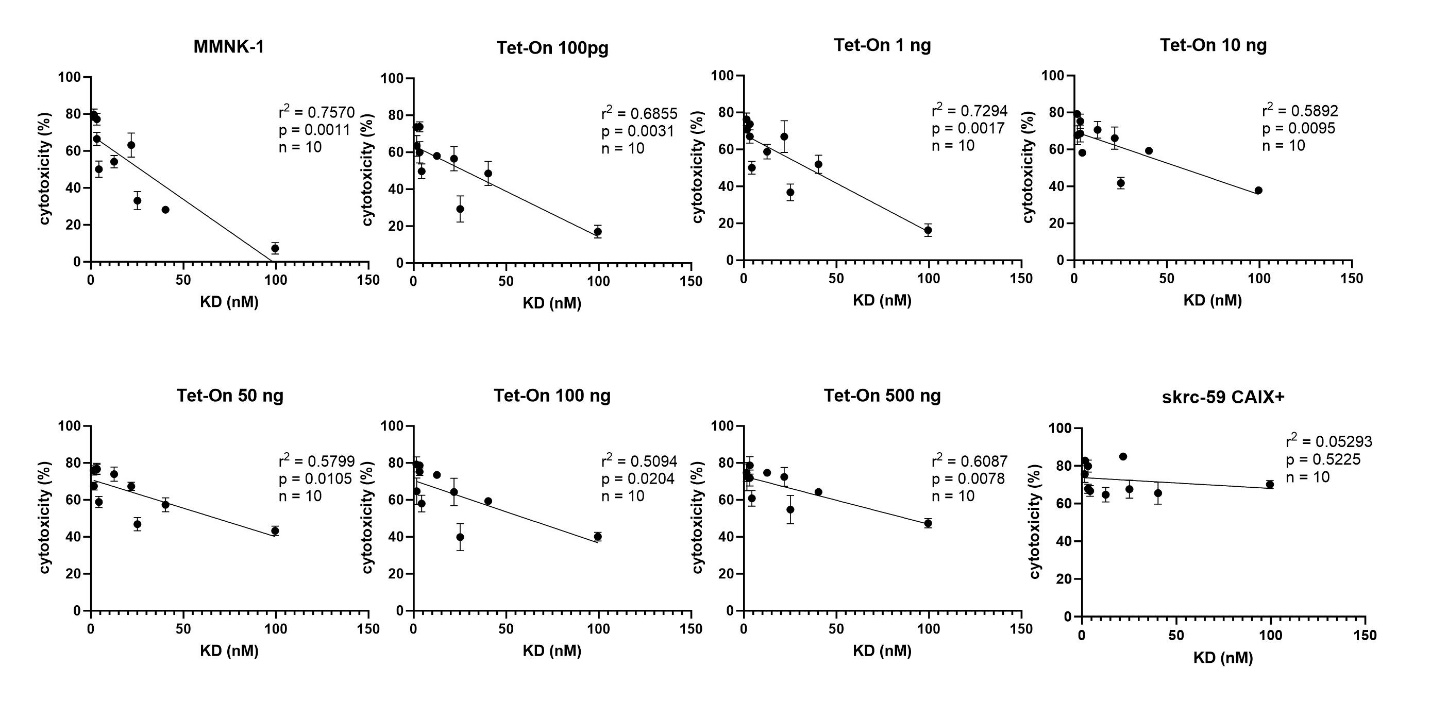


**Supplementary Figure 3. The correlation between cytotoxicity of fine-tuned CAR-T cells and KD value of scFvs.** Supplementary Figure 2 was regraphed by using cytotoxicity of ten anti-CAIX CAR-T cells that developed by Xu et al (G119, G10, G106, G36, G57, G45, G40, G27, G125, G9) and the corresponding KD value of scFvs shown in Figure 2C. Each dot here represents one CAR construct. Linear regression was performed on each plot. Parameters (r^2^, p, n) are shown in the plots. All data with error bars are presented as mean ± SD. *P* values are defined by unpaired two-tailed t-tests (∗p < 0.05; ∗∗p < 0.01; ∗∗∗p < 0.001; and ∗∗∗∗p < 0.0001).


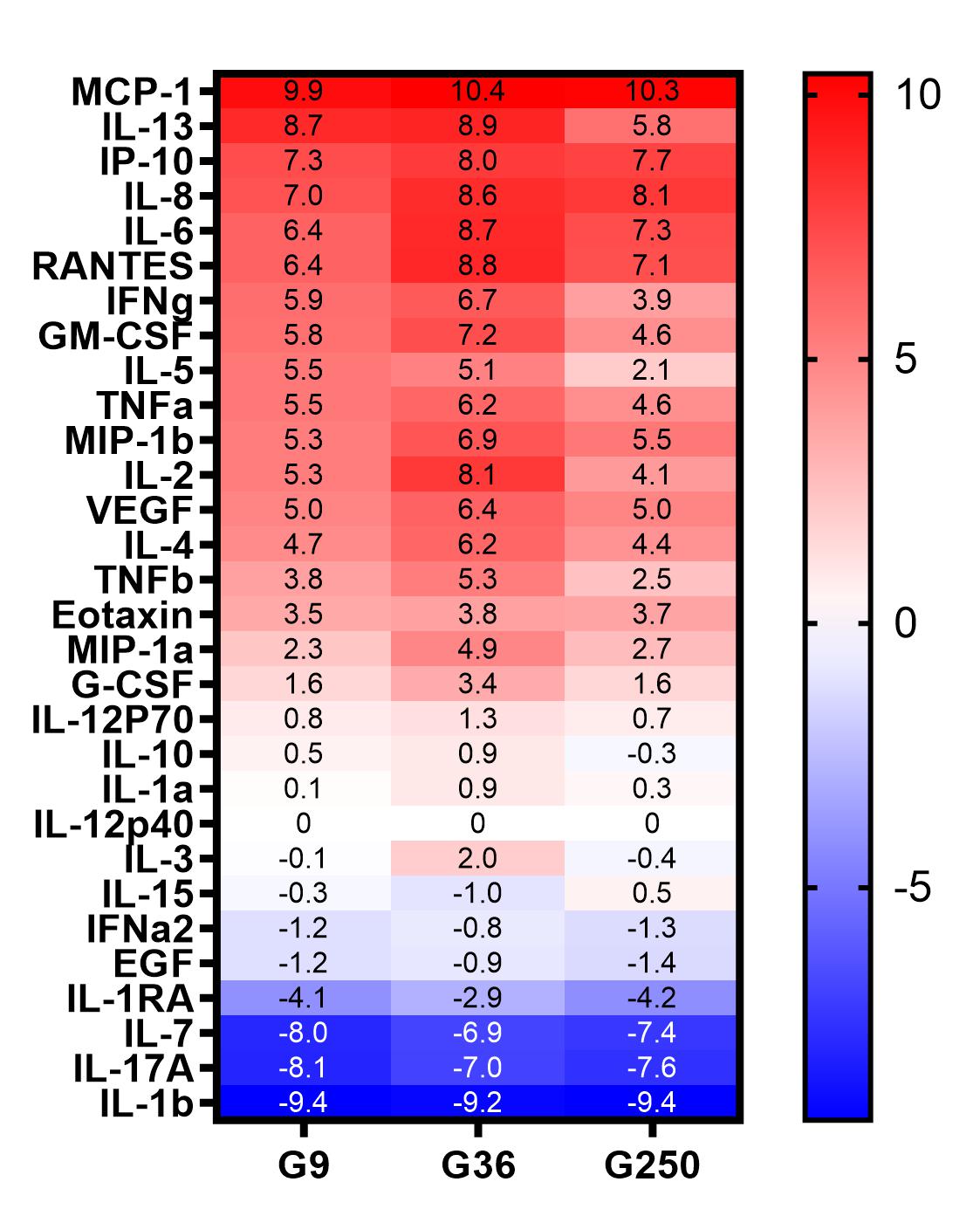


**Supplementary Figure 4. Heatmap of log2 fold change of cytokine and chemokine in the supernatant.** Cytokine profiling was performed via Luminex using supernatant collected on Day 6 from PDOTS (51321216 RCC sample) and CAR-T co-cultures. Eotaxin, EGF, GM-CSF, IFN-γ, IL-10, IL-12/p70, IL-13, IL-15, IL-17α, IL-1Rα, IL-1β, IL-2, IL-3, IL-4, IL-5, IL-7, IL-8, IP-10, MCP-1, MIP-1α, MIP-1β, RANTES, TNFα, TNFβ, VEGF, IL-6, G-CSF, IL-1α, IL-12/p40, IFN-α2 were quantified.


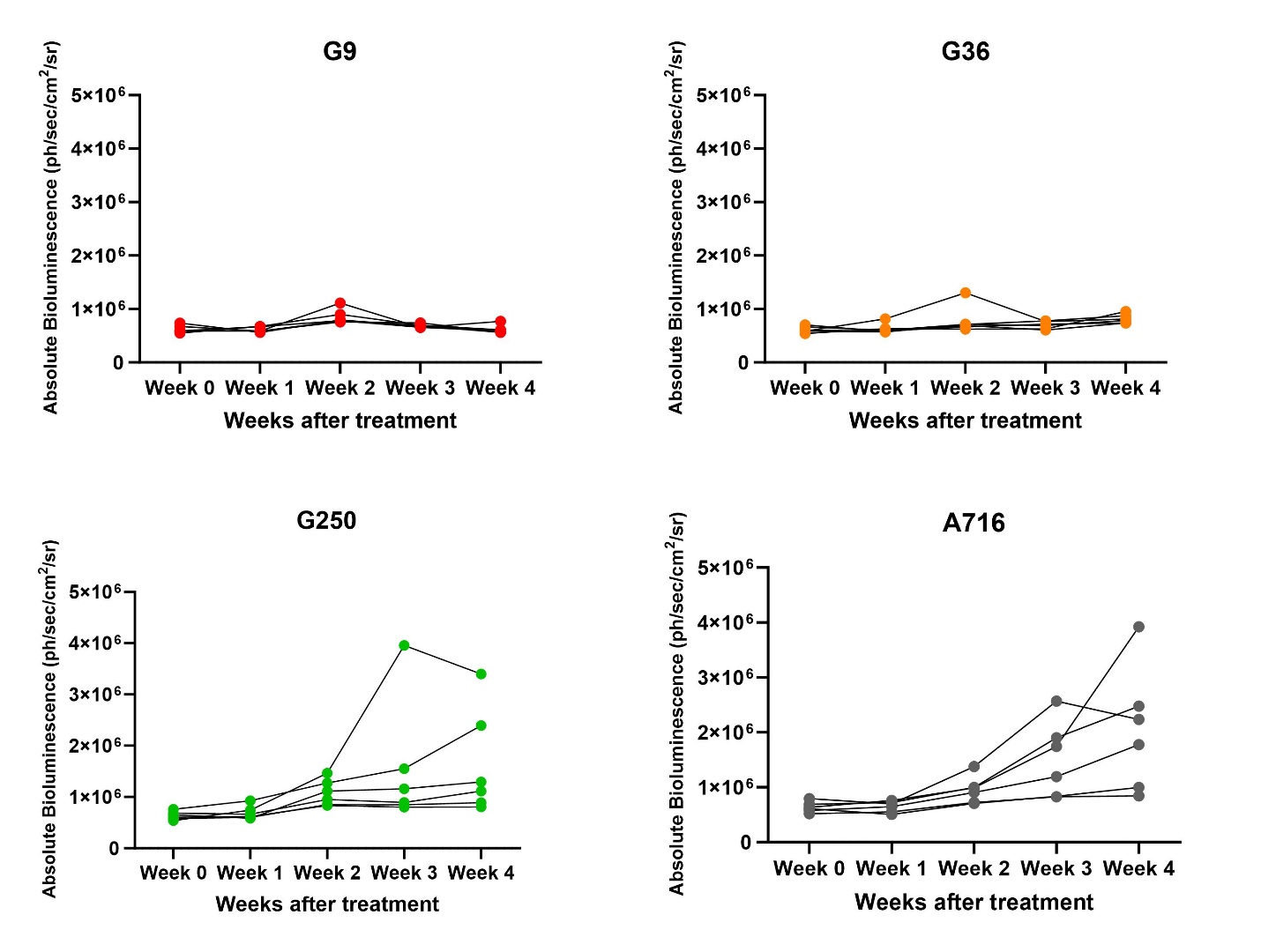


**Supplementary Figure 5. Tumor growth curve of each individual mouse treated with one million CD4:CD8=2:1 G9 (pink), G36 (orange), G250 (green) or A716 (grey) CAR-T cells.** BLI was performed on Day 0, Day 7, Day 14, Day 21 and Day 28 after CAR-T infusion.

**Supplementary Figure 6. Tumor weight of each individual mouse treated with one million CD4:CD8=2:1 G9 (pink), G36 (orange), G250 (green) or A716 (grey) CAR-T cells.** Tumor tissues were collected on Day 28 after CAR-T infusion. All data with error bars are presented as mean ± SD. *P* values are defined by unpaired two-tailed t-tests (∗p < 0.05; ∗∗p < 0.01; ∗∗∗p < 0.001; and ∗∗∗∗p < 0.0001).

**Supplementary Figure 7. Body weight of mice treated with one million CD4:CD8=2:1 G9 (pink), G36 (orange), G250 (green) or A716 (grey) CAR-T cells.** All data with error bars are presented as mean ± SD. *P* values are defined by unpaired two-tailed t-tests (∗p < 0.05; ∗∗p < 0.01; ∗∗∗p < 0.001; and ∗∗∗∗p < 0.0001).

**
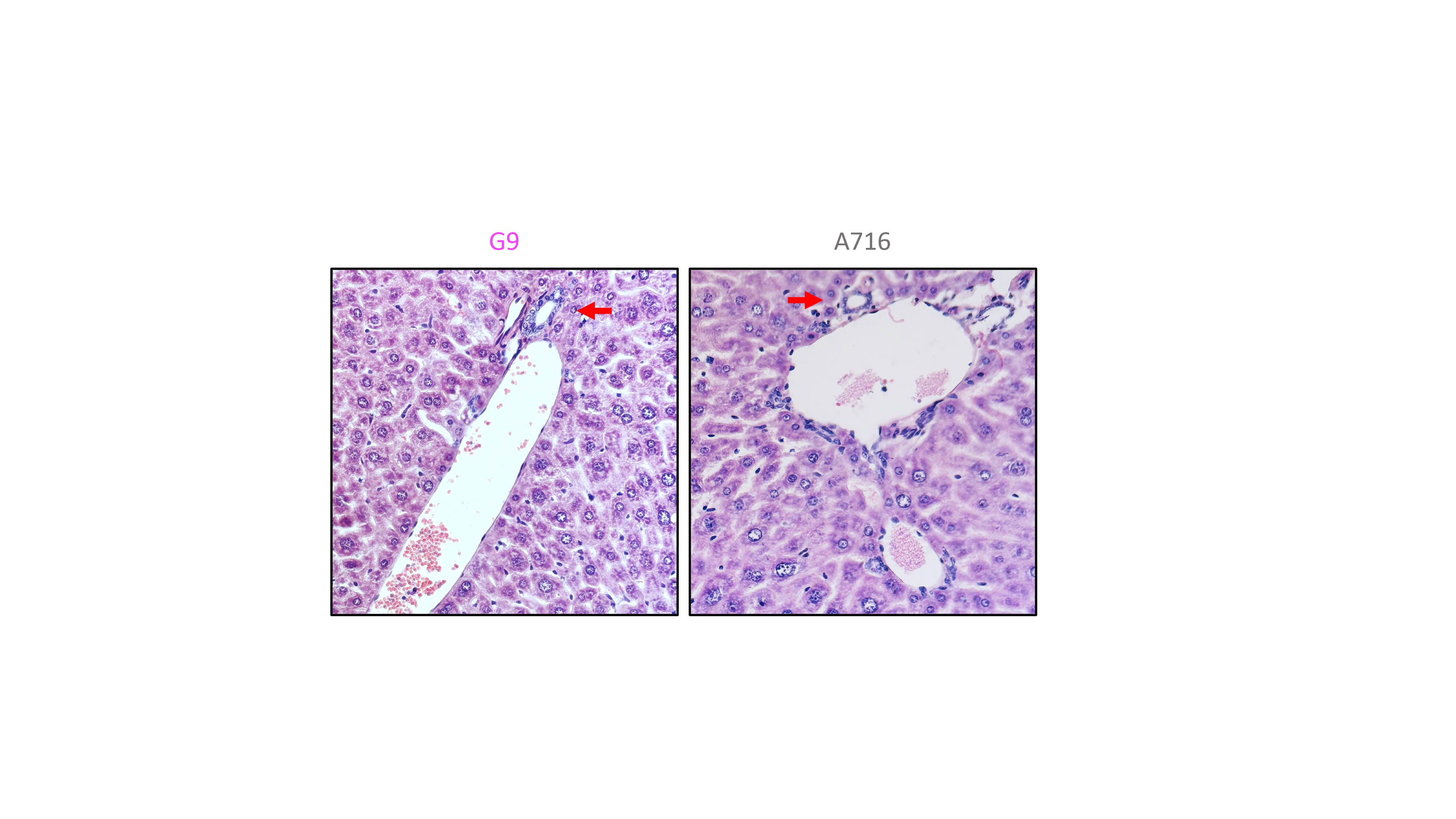
**

**Supplementary Figure 8. H&E staining of portal triad from the mice treated with one million CD4:CD8=2:1 G9 (pink) or A716 (grey) CAR-T cells.** The red arrow indicates the bile duct within portal triad. No pathological changes were observed in the bile duct treated with G9 compared to the one treated with A716.

**Supplementary Tables**

| scFv-Fc | KD (nM) |
| --- | --- |
| G119 | 1.49 |
| G10 | 1.62 |
| G37 | 1.89 |
| G106 | 3.20 |
| G36 | 3.22 |
| G39 | 3.43 |
| G57 | 4.25 |
| G45 | 12.50 |
| G40 | 21.78 |
| G27 | 25.12 |
| G6 | 25.90 |
| G125 | 40.32 |
| G9 | 99.58 |

**Table S1. KD values of anti-CAIX scFv-Fcs ^20^.**

**References**

20. Xu, C., Lo, A., Yammanuru, A., Tallarico, A. S., Brady, K., Murakami, A. *et al.* Unique biological properties of catalytic domain directed human anti-CAIX antibodies discovered through phage-display technology.PLoS One. 2010 Mar 10; **5**: e9625, doi:10.1371/journal.pone.0009625.PMC2835754
